# Effect of nitrogen, phosphorus and potassium fertilization management on soil properties and leaf traits and yield of *Sapindus mukorossi*

**DOI:** 10.3389/fpls.2024.1300683

**Published:** 2024-03-11

**Authors:** Juntao Liu, Dongnan Wang, Xiaoli Yan, Liming Jia, Na Chen, Jiajia Liu, Pengli Zhao, Ling Zhou, Qiuli Cao

**Affiliations:** ^1^ Key Laboratory of Silviculture and Conservation of the Ministry of Education, College of Forestry, Beijing Forestry University, Beijing, China; ^2^ State Key Laboratory of Efficient Production of Forest Resources, Beijing, China; ^3^ National Innovation Alliance of Sapindus Industry, Beijing Forestry University, Beijing, China; ^4^ Key Laboratory of Sustainable Forest Ecosystem Management-Ministry of Education, School of Forestry, Northeast Forestry University, Harbin, China; ^5^ College of Forestry, Fujian Agriculture and Forestry University, Fuzhou, China

**Keywords:** *Sapindus mukorossi*, rational fertilization, soil properties, leaf traits, yield, factor analysis, path analysis

## Abstract

Rational fertilization is the main measure to improve crop yield, but there are differences in the optimal effects of nitrogen (N), phosphorus (P) and potassium (K) rationing exhibited by the same crop species in different regions and soil conditions. In order to determine the optimum fertilization ratio for high yield of *Sapindus mukorossi* in western Fujian to provide scientific basis. We carried out the experimental design with different ratios of N, P and K to investigate the effects of fertilization on the yield. and leaf physiology of *Sapindus mukorossi*and soil properties. Results showed that the yield of *Sapindus mukorossi* reached the highest value (1464.58 kg ha^-1^) at N_2_P_2_K_2_ treatment, which increased to 1056.25 kg ha^-1^ compared with the control. There were significant differences in the responses of soil properties and leaf physiological factors to fertilization treatments. Factor analysis showed that the integrated scores of soil factors and leaf physiological characteristic factors of *Sapindus mukorossi* under N_2_P_2_K_2_ fertilization treatment were the highest, which effectively improved the soil fertility and leaf physiological traits. The yield of *Sapindus mukorossi* showed a highly significant linear positive correlation with the integrated scores (r=0.70, *p*<0.01). Passage analysis showed that soil available nitrogen content, organic carbon content, and leaf area index were the key main factors to affect the yield. RDA showed that soil organic carbon and available phosphorus were the most important factors to affect leaf physiological traits. We recommend that the optimum fertilization ratio of *Sapindus mukorossi* was 0.96Kg N, 0.80Kg P and 0.64Kg K per plant. Reasonable fertilization can improve soil fertility and leaf physiological traits, while excessive fertilization has negative effects on soil fertility, leaf physiology and yield. This study provides theoretical support for scientific cultivation of woody oil seed species.

## 1 Introduction

With the increasing global demand for energy, bioenergy species have received widespread attention as an emerging and promising energy resource (Xu et al., 2023). As an energy species, *Sapindus mukorossi*, distributed in tropical and subtropical regions of China, is widely used in biomedicine, bioenergy and cosmetics (Zhao et al., 2019; Wang et al., 2021; Xu et al., 2021; Liu et al., 2022). The whole plant of *Sapindus mukorossi*, including its fruits, roots, bark and leaves, has been used widely in traditional medicine in China (Xu et al., 2023). For instance, Saponin, extracted from the pericarp (protein content of about 22%) of *Sapindus mukoross*, is become a good foaming agent and pesticide. And the kernel of the soap berries contains about 40% fatty acids, which can be used to produce biodiesel and advanced lubricants. It is also a woody oilseed species that has been promoted for use in recent years (Xu et al., 2022). Only in Fujian Province in Chinese, the planting area reaches 20000 ha, which is one of the main production areas. However, under the previous planting mode of rough management, the yield and quality of *Sapindus mukorossi* were low (Gao et al., 2018, 2022). Therefore, it is a key issue to improve the yield of *Sapindus mukorossi*. Previous studies have found that scientific fertilization is the most direct and effective method to improve the yield, except good seed selection, density control, and shaping and pruning mixed plantation (Gao et al., 2018; Zhang et al., 2019; Zhou et al., 2020; Uçgun and Altindal, 2021; Liu et al., 2022; Xiang et al., 2022). It has been shown that nitrogen (N), phosphorus (P) and potassium (K) fertilization has a significant effect on soil physical and chemical properties of cash crops, and fertilization promotes root and inter-root microbial exudation, the effectiveness of soil fast-acting nutrients that can be directly absorbed and utilized by the plant, which ultimately affects the yield (Brunetto et al., 2015; Liu et al., 2021; Li et al., 2022). However, current research on the effect of fertilization on yield in *Sapindus mukorossi* focuses on the role of single nutrient fertilizers, while the interaction between N, P, and K on how it affects the yield of *Sapindus mukorossi* is yet to be studied.

The application of fertilizers in different ratios between N, P and K is a balanced fertilization method. Balanced fertilization refers to the application technology of reasonable fertilizer dosage and ratio based on the fertilizer demand characteristics of crops, soil fertilizer supply performance and fertilizer dosage to maintain nutrient balance between the proposed fertilizer dosage and ratio. For example, Zhang et al. (2023) showed that N, P and K fertilizers have different effects on fruit yield and quality, of which K fertilizer is the main factor affecting yield and quality, and reasonable fertilization can significantly improve the yield and fruit quality of blueberries (*Vaccinium virgatum*). Yin et al. (2018) found that the crop of mung bean (*Vigna radiata*) has a significant increase with N, P and K fertilizers. Relevant studies have shown that moderate nitrogen, phosphorus, and potassium fertilization can effectively increase the nutrient content of plant leaves and soil, thereby increasing crop yield and quality (Zhang et al., 2020; Wan et al., 2021; Zhang et al., 2022). Proper fertilizer management can maximize lettuce biomass production, but excessive fertilizer supply can lead to disease episodes that impair leaf and root development and nitrate accumulation (Shi et al., 2021). In addition, excessive fertilizers will also cause soil acidification leading to severe soil nutrient imbalance, which induces leaf nutrient imbalance and ultimately affects yield and economic efficiency (Raliya et al., 2017; Xue et al., 2020). Therefore, the proportions of N, P, and K are often unbalanced due to the lack of scientific ratios in actual production, which affects the uptake and utilization of nutrients by plants, and reduces yield and quality, and increases the risk of nutrient loss and environmental pollution (Rahman and Zhang, 2018). Therefore, an appropriate ratio of N, P and K can significantly promote plant growth and reduce the usage of different fertilizers relatively (Yang et al., 2020).

Fertilizer application can directly or indirectly change the physical and chemical properties of soil, thus altering soil productivity (Li et al., 2020; Ren et al., 2020; Hou et al., 2023). Li et al. (2019) found that fertilizer application had a significant effect on soil available nutrients and yield of citrus, but soil available nutrients and yield showed an increasing and then a decreasing trend. Reasonable combination of N, P, and K application can improve soil nutrients and fruit yield (Mete et al., 2015; Zhang et al., 2020). Whereas, excessive fertilization will result in lower N, P and K content in the soil (Li et al., 2019). Meanwhile, fertilization has significant effects on leaf functional traits (Bassi et al., 2018), such as chlorophyll content (Xiong et al., 2015), non-structural carbohydrates (Sun et al., 2020), and leaf nutrients (Liu et al., 2021). For example, Sardans et al. (2017) found that in temperate forests, fertilization increased leaf nitrogen content, while the effect on phosphorus content was not significant (Sardans et al., 2017). Bassaco et al. (2018) found that the addition of N, P, and K fertilizers had a boosting effect on specific leaf area, soluble sugar content, protein content, and photosynthetic rate of eucalyptus (*Eucalyptus urograndis*) (Bassaco et al.2018), however, over-fertilization can negatively affect the morphological and chemical traits of leaves. For example, excessive nitrogen fertilization can have a negative effect on leaf physiological traits of banana (*Musa nana*) and reduce soil nutrient content (Sun et al., 2020). In conclusion, under- or over-fertilization can have an effect on soil physical and chemical properties, leaf traits, and yield of plants (Yang et al., 2020; Zhang et al., 2020).

In this study, we used 6-year-old *Sapindus mukorossi* as a test material, and adopted the “3414” fertilizer formulation method, focusing on which fertilizer rate is the most effective and which soil properties and leaf traits play a key role in yield. Three hypotheses were formulated as follows: (1) Is yield highest at the highest N, P and K fertilization rate? (2) Determine the major soil and leaf factors that influence *Sapindus mukorossi* yield; (3) Which leaf traits influence soil nutrients under different NPK fertilization treatments. Meanwhile, we explore *Sapindus mukorossi* yield and the combined scores of soil and leaf factors to estimate the optimal fertilization ration. This study can further determine the optimal ratio and mechanism of N, P and K fertilization of *Sapindus mukorossi* in western Fujian, and provide theoretical support for scientific improvement of *Sapindus mukorossi* yield.

## 2 Materials and methods

### 2.1 Study site

The study site was located in Jianning County, Sanming City, Fujian Province (116°47′20″E, 26°40′3″N), with an average annual temperature of 17.0°C, an average annual rainfall of 1,792 mm, and a relative humidity of 84%. The soil of the test site was sandy clay loam, with soil organic carbon content of 7.75 g kg^-1^, total nitrogen content of 1.40 g kg^-1^, total phosphorus content of 0.36 g kg^-1^, total potassium content of 27.95 g kg^-1^, fast-acting potassium content of 48.16 mg kg^-1^, effective phosphorus content of 1.32 mg kg^-1^, and alkaline dissolved nitrogen content of 36.01 mg kg^-1^.The raw material forest of *Sapindus mukorossi* was the asexual line of ‘Yuanhua’, with an average height of 2.38 m, an average diameter of 6.97 cm, and an average crown width of 2.4 m × 1.4 m. The average height was 2.38 m, with an average diameter of 6.97 cm. The average tree height was 2.38m, the average diameter was 6.97cm, and the average crown width was 2.4m×2.2m.

### 2.2 Experimental design

We conducted a field trial using a combination of different levels of nitrogen (N), phosphorus (P), and potassium (K) (0 level: 0kg/hm^2^ of N, P, and K; 1 level: 300kg/hm^2^ of N, 250kg/hm^2^ of P, and 200kg/hm^2^ of K; 2 level: 600kg/hm^2^ of N, 500kg/hm^2^ of P, and 400kg/hm^2^ of K; and 3 level: 900kg/hm^2^ of N, 750kg/hm^2^ of P, and 600kg/hm^2^ of K) fertilizers in combination with unfertilized control treatments, Fertilizer application rates are shown in **Table 1**, and isolated rows were set up between treatment plots. Fertilizer was applied three times throughout the year, April 10 (flowering fertilizer, accounting for 30% of the total), July 20 (strong fruit fertilizer, accounting for 30% of the total), November 1 (post-harvest fertilizer, accounting for 40% of the total), using furrow fertilization method to apply fertilizer, according to the fertilizer dosage mixed into the application of the soil immediately after mulching, and other maintenance and management measures are the same as those of the control group. The test fertilizers were urea (containing N 46.0%) as the only source of N, calcium superphosphate (containing P_2_O_5_ 12.0%) as the only source of P, and potassium sulfate (containing K_2_O 60.0%) as the only source of K.

**Table 1:** Combination of factors and levels in each treatment.

Processing number	Fertilization level (kg hm^-2^)		
	N	P	K
N_0_P_0_K_0_	0	0	0
N_0_P_2_K_2_	0	500	400
N_1_P_2_K_2_	300	500	400
N_2_P_0_K_2_	600	0	400
N_2_P_1_K_2_	600	250	400
N_2_P_2_K_2_	600	500	400
N_2_P_3_K_2_	600	750	400
N_2_P_2_K_0_	600	500	0
N_2_P_2_K_1_	600	500	200
N_2_P_2_K_3_	600	500	600
N_3_P_2_K_2_	900	500	400
N_1_P_1_K_2_	300	250	400
N_1_P_2_K_1_	300	500	200
N_2_P_1_K_1_	600	250	200

N_0_, N_1_, N_2_, and N_3_ denote four N applications, 0, 300, 600, and 900 kg.hm^-2^, P_0_, P_1_, P_2_, and P_3_ denote four phosphorus applications, 0, 250, 500, and 750 kg hm^-2^, and K_0_, K_1_, K_2_, and K_3_ denote four potassium applications, 0, 200, 400, and 600 kg hm^-2^, respectively.

### 2.3 Plant and soil sampling

Soil sampling: On August 20, 2023, three randomly selected sampling points in each plot, respectively, 0-20cm soil samples, soil samples collected were divided into two parts, one part of the self-sealing bag to bring back to the laboratory to naturally dry 2mm sieve, for the determination of soil chemical properties; the other part of the 50ml centrifugal tubes, with an ice box to save the soil samples, brought back to the laboratory at a low temperature of 4 °C preservation for soil microbial biomass. Soil organic carbon was determined by potassium dichromate oxidation-external heating method, available nitrogen (AN) was determined by alkaline dissolution diffusion method, soil effective phosphorus was determined by molybdenum antimony colorimetric method, Soil available potassium was determined by flame photometric method, and pH was measured by using a mixture of soil and water (1:2.5) (Klotz et al., 2023; Lin et al., 2023). Microbial biomass carbon (MBC) and nitrogen (MBN) were measured by chloroform fumigation-K_2_SO_4_ extraction method assay (Borah et al., 2023; de Souza et al., 2023).

Leaf sampling: collected at the same time as soil samples. A standard branch was selected in the middle canopy of each tree and 20 well-developed, well-rounded leaves were collected from it. The upper part of the functional leaves on the main stem that were well developed and fully expanded without disease were selected and the middle and upper parts were sampled. The leaves were killed at 105°C for 30min, dried at 55°C until constant weight, crushed and sieved to determine the leaf nitrogen (N), phosphorus (P) and potassium (K) content. Leaf nitrogen content was determined by Kjeldahl method; leaf phosphorus content was determined by Molybdenum antimony colorimetric method; leaf potassium content was determined by flame photometric method (Yang et al., 2021).

Chlorophyll SPAD measurement: chlorophyll content was measured using a SPAD-502 portable chlorophyll meter prior to leaf sampling. Three SPAD readings were taken on the same leaves, eight plants per plot, and the average SPAD reading was calculated.

Leaf area index (LAI): The canopy leaf area index (LAI) of *Sapindus mukorossi* was determined by using the LAI-2200 plant canopy analyzer of Li-Cor, U.S.A. The horizontal direction of the instrument’s sensor probe was kept in the same level with the ground when the measurement was carried out.

Non-structural carbohydrates: soluble sugar and starch contents were determined by anthrone colorimetric method (Hajihashemi et al., 2020).

Yield determination: sapodilla was harvested at maturity. *Sapindus mukorossi* yield was calculated as the average yield of a single plant by weighing the weight through electronic scale after harvesting.

### 2.4 Statistical analyses

One-way grouped data were analyzed by analysis of variance (ANOVA) and multiple comparisons by the new complex extreme variance method (Duncan) using SPSS 21.0. The effects of different fertilization treatments on soil properties and physiological characteristics of sapodilla leaves were comprehensively evaluated by factor analysis. In factor analysis, principal component analysis (PCA) was used to extract factors from selected variances. It attempts to explain complex variance with a minimum number of factors that explain the variance better. Factors were extracted in the order of the weight of each factor. Factors with eigenvalues ≥ 1 change in the data were retained (Sun et al., 2020). Direct and indirect effects of soil and foliage factors on the yield of *Sapindus mukorossi* were evaluated using pathway analysis. The direct effects could be obtained from the direct path coefficients. Indirect effects were calculated from the equation of path coefficient × correlation coefficient. The relationship between leaf physiology and soil factors was analyzed using the R language Vegan package. First, the raw data were subjected to detrended correspondence analysis (DCA) to determine whether they were suitable for using the single-peak model (CCA) or the linear model (RDA). The results showed that the data of this experiment were analyzed using the redundancy of linear model. Important soil factors affecting the overall variation in leaf physiological attributes were investigated.

## 3 Results

### 3.1 Effect of different fertilization treatments on the yield of *Sapindus mukorossi*

**Figure 1:**
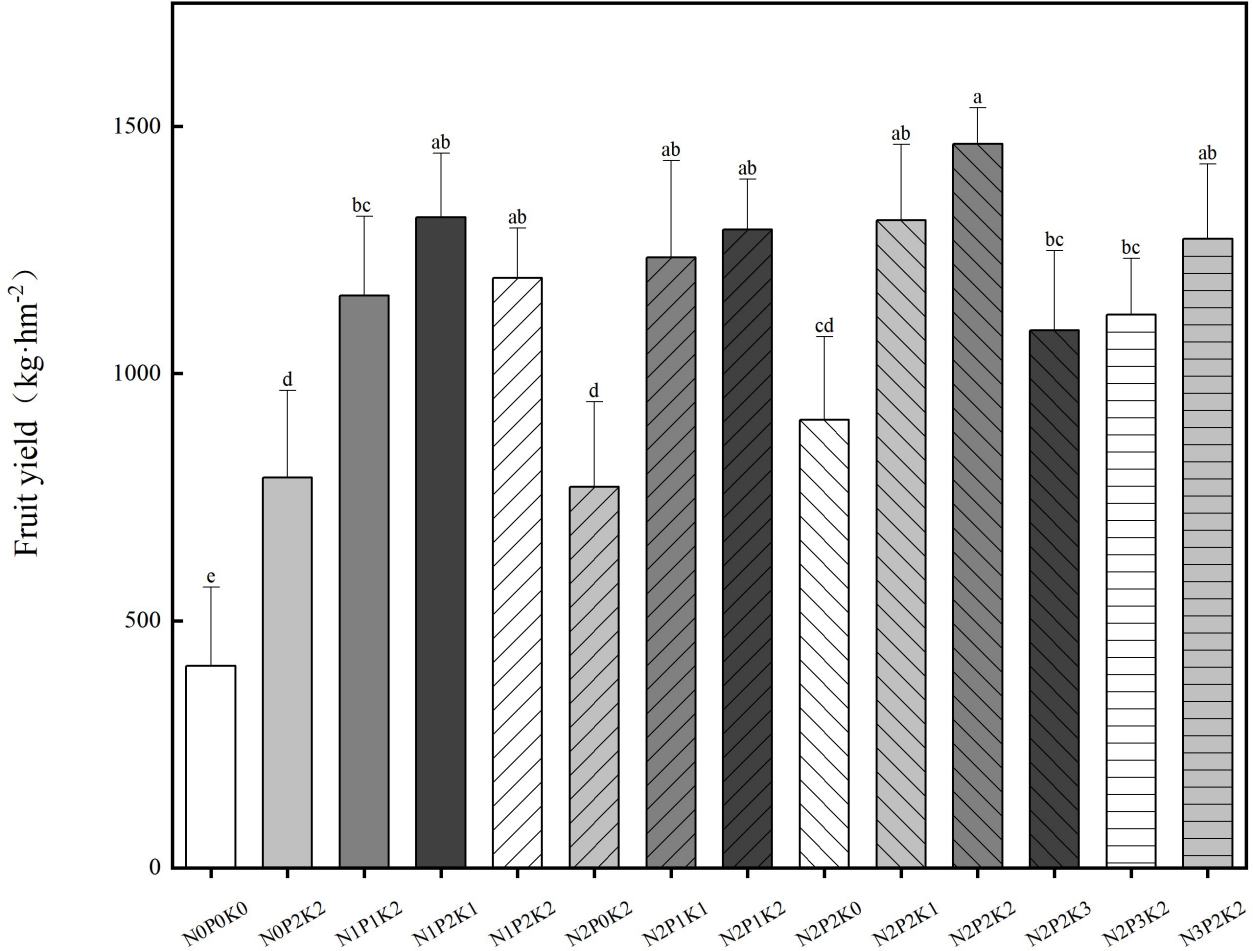
Effects of different nitrogen, phosphorus and potassium fertilizers on the yield of *Sapindus mukorossi*. Different letters in the same column represent significant differences in sapodilla at p < 0.05 for different fertilization treatments (Tukey's test).

Different fertilization treatments significantly affected the yield of *Sapindus mukorossi* (**Figure 1**). The highest value of *Sapindus mukorossi* yield (1464.58 kg ha^-1^) was achieved at N_2_P_2_K_2_ treatment, which increased by 258.67% as compared to control.

At the P_2_K_2_ level, the *Sapindus mukorossi* yield under each fertilization treatment showed a trend of increasing and then decreasing with the addition of N application, and there was no significant difference between fertilization treatments of N_2_P_2_K_2_ and N_3_P_2_K_2_. At the N_2_K_2_ level, the *Sapindus mukorossi* yield under each fertilization treatment showed a trend of increasing and then decreasing with the addition of P application, and there was a significant difference between treatments of N_2_P_2_K_2_ and N_2_P_3_K_2_. At the N_2_P_2_ level, the yield of *Sapindus mukorossi* under all fertilization treatments showed a trend of increasing and then decreasing with the addition of K, and there was a significant difference between N_2_P_2_K_2_ and N_2_P_2_K_3_ treatment.

### 3.2 Effect of different fertilizer treatments on soil properties

There were significant differences in the nutrient characteristics of *Sapindus mukorossi* soils with different fertilization treatments (**Table 2**). Soil organic carbon, available phosphorus, available potassium, available nitrogen, microbial nitrogen, and microbial carbon contents were higher than the control except for soil pH. Compared with the control, the organic carbon, alkaline dissolved nitrogen, microbial nitrogen, available phosphorus, and available potassium contents of *Sapindus mukorossi* soils in fertilizer treatments were increased by 14.17%~52.53%, 3.36%~40.37%, 11.84%~46.50%, respectively, 118.58% to 407.08%, and 5.39% to 39.69%.

**Table 2:** Soil properties under different fertilization treatments.

Treatment	SOC content (g.kg^-1^)	Available P content (mg.kg^-1^)	Available K content (mg.kg^-1^)	Available N content (mg.kg^-1^)	pH	MBN content(mg.kg^-1^)	MBC content(mg.kg^-1^)
N_0_P_0_K_0_	11.08 ± 0.75h	1.13 ± 0.07h	81.79 ± 2.90g	90.21 ± 6.51f	5.02 ± 0.04a	10.30 ± 1.12e	124.89 ± 6.30f
N_0_P_2_K_2_	12.76 ± 0.53fg	2.47 ± 0.14g	93.57 ± 1.25cdef	93.49 ± 0.99f	5.09 ± 0.02a	12.18 ± 0.36cd	154.38 ± 13.61cd
N_1_P_2_K_2_	13.74 ± 0.81def	2.97 ± 0.08g	104.36 ± 1.54abc	105.59 ± 3.82cd	4.82 ± 0.07c	14.31 ± 0.78ab	133.19 ± 4.15ef
N_2_P_0_K_2_	13.52 ± 0.68defg	2.80 ± 0.45g	92.68 ± 4.55defg	97.36 ± 4.17ef	5.04 ± 0.02a	11.79 ± 1.91de	136.26 ± 8.41ef
N_2_P_1_K_2_	14.21 ± 0.67d	4.32 ± 0.58cde	86.17 ± 9.31fg	113.92 ± 5.86bc	4.84 ± 0.07c	12.36 ± 0.53cd	154.20 ± 8.77cd
N_2_P_2_K_2_	15.41 ± 0.68bc	5.29 ± 0.22ab	107.46 ± 6.89ab	126.66 ± 5.42a	4.82 ± 0.03cd	13.85 ± 1.35abc	173.57 ± 4.57ab
N_2_P_3_K_2_	14.05 ± 0.78de	5.73 ± 0.26a	88.81 ± 3.15efg	95.77 ± 5.23f	4.67 ± 0.05f	14.45 ± 1.12ab	166.46 ± 4.99bc
N_2_P_2_K_0_	12.99 ± 0.37efg	4.46 ± 0.29cd	96.26 ± 4.52cdef	109.63 ± 6.12cd	4.91 ± 0.03b	12.35 ± 0.35cd	167.75 ± 10.5bc
N_2_P_2_K_1_	15.68 ± 0.47b	4.78 ± 0.15bc	90.53 ± 7.27efg	106.3 ± 1.39cd	4.81 ± 0.04cd	13.05 ± 0.93bcd	184.16 ± 9.16a
N_2_P_2_K_3_	14.38 ± 0.34cd	3.84 ± 0.39ef	114.26 ± 10.78a	115.11 ± 3.88b	4.71 ± 0ef	11.52 ± 1.14de	163.58 ± 9.88bc
N_3_P_2_K_2_	13.85 ± 0.67de	4.85 ± 0.29bc	87.99 ± 4.36efg	123.92 ± 2.97a	4.56 ± 0.04h	13.30 ± 0.99abcd	156.85 ± 8.76cd
N_1_P_1_K_2_	16.90 ± 0.29a	3.96 ± 0.29def	98.40 ± 6.33bcde	103.93 ± 2.64de	4.78 ± 0.04cde	15.09 ± 0.88a	169.27 ± 7.73abc
N_1_P_2_K_1_	14.46 ± 0.28cd	3.57 ± 0.16f	94.70 ± 6.86cdef	111.74 ± 6.49bcd	4.80 ± 0.03cd	12.29 ± 0.55cd	183.05 ± 2.94a
N_2_P_1_K_1_	12.65 ± 0.41g	2.77 ± 0.26g	101.91 ± 3.44bcd	112.85 ± 4.27bc	4.74 ± 0.02def	11.72 ± 0.63de	143.59 ± 9.09de

Different lower-case letters indicate significant difference between fertilization treatments at 0.05 level.

At the P_2_K_2_ level, except for pH, all other indexes showed a trend of increasing and then decreasing with the addition of N application, and the difference between fertilized treatments N_2_P_2_K_2_ and N_0_P_2_K_2_ was significant (*p* < 0.05), while there was no difference between available phosphorus, available nitrogen, and microbial nitrogen content with N_3_P_2_K_2_. At the N_2_K_2_ level, soil available phosphorus and microbial nitrogen contents showed a gradual increase with the increase in phosphorus application, and the differences were significant (*p* < 0.05) between N_2_P_2_K_2_ and N_2_P_0_K_2_ treatment. At the N_2_P_2_ level, the trends of soil organic carbon and available nitrogen were similar to those at the P_2_K_2_ level.

One-way ANOVA showed that different N, P and K treatments had significant effects on soil organic carbon, available phosphorus, available potassium, available nitrogen, microbial nitrogen and carbon contents. The results showed that the soil factors had different effects on the response of each fertilization, and the soil nutrient factor indexes increased its soil nutrient content gradually with the increase of fertilizer application. When the fertilizer application reached a certain amount, soil nutrient content reached their peak. But there will exist a decreasing trend, if the fertilizer continued to be increased.

### 3.3 Effect of different fertilization treatments on leaf traits

Different fertilization treatments had significant effects on leaf traits (**Table 3**). The leaf area index, chlorophyll, soluble sugar, starch, nitrogen, phosphorus and potassium contents of *Sapindus mukorossi* leaves under all fertilization treatments increased and improved to different degrees compared with the control.

**Table 3:** Leaf traits of *Sapindus mukorossi* under different fertilization treatments.

Treatment	LAI	Chlorophyll content (SPAD value)	Soluble sugar (g.kg^-1^)	starch (g.kg^-1^)	Nitrogen content (g.kg^-1^)	Phosphorus content (g.kg^-1^)	Potassium content (g.kg^-1^)
N_0_P_0_K_0_	1.08 ± 0.13c	43.91 ± 0.51b	56.50 ± 2.03def	24.47 ± 1.86e	21.65 ± 1.20bcd	1.20 ± 0.15efg	18.61 ± 2.33cd
N_0_P_2_K_2_	1.13 ± 0.07bc	44.03 ± 0.80b	63.86 ± 1.87c	23.53 ± 2.33e	18.52 ± 0.95e	1.18 ± 0.10fg	18.57 ± 1.17cd
N_1_P_2_K_2_	1.20 ± 0.08abc	44.77 ± 1.50ab	54.32 ± 2.79fg	24.65 ± 1.43e	21.16 ± 0.29cd	1.35 ± 0.06cdef	18.93 ± 0.53cd
N_2_P_0_K_2_	1.18 ± 0.04abc	45.12 ± 0.63ab	66.24 ± 2.80bc	26.24 ± 0.56e	22.13 ± 0.75bcd	1.43 ± 0.11cd	19.77 ± 0.75c
N_2_P_1_K_2_	1.24 ± 0.06ab	44.72 ± 0.42ab	53.81 ± 2.67fg	25.58 ± 0.70e	23.21 ± 0.40ab	1.65 ± 0.10ab	21.75 ± 0.74b
N_2_P_2_K_2_	1.26 ± 0.01ab	46.89 ± 0.96a	60.02 ± 2.41d	39.38 ± 1.01a	22.64 ± 1.13abc	1.71 ± 0.09a	20.45 ± 0.53bc
N_2_P_3_K_2_	1.21 ± 0.06abc	43.38 ± 0.96b	68.72 ± 0.78ab	36.21 ± 2.10b	23.55 ± 1.38ab	1.28 ± 0.10defg	18.50 ± 0.66cd
N_2_P_2_K_0_	1.14 ± 0.11abc	44.48 ± 1.13b	56.19 ± 1.24defg	25.77 ± 1.80e	22.42 ± 1.03bc	1.52 ± 0.03bc	18.68 ± 0.58cd
N_2_P_2_K_1_	1.28 ± 0.10a	44.28 ± 0.86b	53.49 ± 2.02fg	29.13 ± 1.13d	20.72 ± 0.91cd	1.27 ± 0.08defg	18.71 ± 0.65cd
N_2_P_2_K_3_	1.22 ± 0.04abc	45.37 ± 1.26ab	59.71 ± 0.47de	32.20 ± 0.98c	23.32 ± 0.78ab	1.25 ± 0.01defg	19.01 ± 0.75cd
N_3_P_2_K_2_	1.19 ± 0.02abc	44.35 ± 0.94b	70.17 ± 1.64a	26.24 ± 1.96e	24.50 ± 1.06a	1.29 ± 0.11defg	24.47 ± 0.93a
N_1_P_1_K_2_	1.17 ± 0.05abc	44.89 ± 2.49b	52.46 ± 1.73g	31.17 ± 0.42cd	20.31 ± 0.65d	1.39 ± 0.15cde	19.31 ± 0.53cd
N_1_P_2_K_1_	1.27 ± 0.03ab	43.87 ± 1.27b	57.43 ± 1.12def	29.41 ± 1.70d	21.13 ± 0.76cd	1.14 ± 0.12g	17.62 ± 1.32d
N_2_P_1_K_1_	1.24 ± 0.07ab	44.99 ± 1.43ab	55.88 ± 3.27efg	25.59 ± 1.59e	22.25 ± 1.96bcd	1.28 ± 0.05defg	18.46 ± 1.24cd

Different letters in the same column represent significant differences in sapodilla at p < 0.05 for different fertilization treatments (Tukey's test).

At the P_2_K_2_ level, leaf area index, chlorophyll, starch, phosphorus content indicators under each fertilization treatment showed a trend of increasing and then decreasing with the addition of nitrogen application, and reached the highest value in the N_2_P_2_K_2_ treatment, and soluble sugar, nitrogen, and potassium content showed a gradual increase with the addition of nitrogen application; at the N_2_K_2_ level, leaf area index, chlorophyll, starch, phosphorus content, and potassium content indicators of *Sapindus mukorossi* leaves increased and improved to different degrees with the addition of nitrogen application and potassium content indicators all showed an increasing and then decreasing trend with the addition of nitrogen application, and soluble sugar content showed a gradual increasing trend with the addition of nitrogen application; at the N_2_P_2_ level, leaf area index, chlorophyll, soluble sugar, starch, nitrogen, phosphorus and potassium content all showed an increasing and then decreasing trend with the addition of nitrogen application.

One-way ANOVA showed that fertilizer application had a significant effect on soluble sugar, starch, total potassium, total nitrogen, total phosphorus content, and yield of *Sapindus mukorossi* leaves, and there was no significant difference on leaf area index and chlorophyll content. In conclusion, leaf physiological traits were reached the highest value in N_2_P_2_K_2_ treatment, whatever applying different levels of N, P and K fertilizer.

### 3.4 Comprehensive evaluation of different fertilization treatments on soil factors and leaf traits

Soil factors and leaf physiological traits under each fertilization treatment were attributed into five factors by factor analysis with a cumulative contribution of 85.47% (**Table 4**). Factor 1 contributed 39.12% to soil organic carbon (0.337), soil available nitrogen (0.334), and available phosphorus (0.369). The fertilizer combination with the highest score (4.49) for factor 1 was N_2_P_2_K_2_. This indicates that N_2_P_2_K_2_ was the most effective in improving the soil factors of sapodilla. Factor 2 contributed 17.01% with higher absolute factor loadings of leaf nitrogen and potassium content. Factor 2 was named as leaf nutrient factor. N_1_P_1_K_2_ fertilization combination had the highest factor 2 score (2.45). This indicated that N_1_P_1_K_2_ treatment was dominant in increasing the nutrient content of sapodilla leaves. Factor 3 contributed 14.00% positive factor loading for chlorophyll content (0.56) and was named as photosynthetic pigment factor. N_2_P_2_K_2_ treatment had the highest factor 3 score (2.25) indicating that N_2_P_2_K_2_ treatment was the most effective in improving photosynthetic physiological traits of leaves. Factor 4 contributed 8.21% and had the highest loading of soil quick potash (0.447) and was named as soil potassium nutrient factor. N_2_P_2_K_3_ treatment had the highest score (2.34) for factor 4 indicating that N_2_P_2_K_3_ treatment was the most effective in improving soil quick potash. Factor 5 contributed 7.12% with high absolute factor loadings of leaf soluble sugar and starch and factor 5 was named as sucrose metabolism factor. N_2_P_1_K_2_ treatment had the highest score (2.34) for factor 5 which indicated that N_2_P_1_K_2_ treatment was dominant in increasing sucrose metabolism in *Sapindus mukorossi*.

**Table 4:** Composite scores under different fertilization treatments.

Treatment	Factor 1 score (order)	Factor 2 score (order)	Factor 3 score (order)	Factor 4 score (order)	Factor 5 score (order)	Integrated score (order)
N_0_P_0_K_0_	-5.33 (14)	-0.81 (11)	0.38 (8)	-0.07 (7)	-0.09 (9)	-1.81 (14)
N_0_P_2_K_2_	-3.61 (13)	1.11 (4)	-0.48 (9)	-0.52 (9)	-0.8 (11)	-1.19 (13)
N_1_P_2_K_2_	-0.60 (10)	0.73 (5)	0.91 (6)	0.09 (5)	0.28 (6)	0.18 (8)
N_2_P_0_K_2_	-1.79 (12)	-0.87 (12)	0.98 (4)	-0.68 (11)	-1.15 (12)	-0.82 (12)
N_2_P_1_K_2_	0.86 (7)	-1.28 (13)	0.96 (5)	-1.56 (14)	1.71 (1)	0.19 (7)
N_2_P_2_K_2_	4.49 (1)	0.28 (6)	2.25 (1)	-0.56 (10)	-1.42 (13)	1.64 (1)
N_2_P_3_K_2_	1.13 (5)	-0.77 (10)	-3.29 (14)	0.12 (4)	-1.67 (14)	-0.63 (11)
N_2_P_2_K_0_	-0.47 (9)	-0.04 (8)	0.58 (7)	-0.71 (12)	0.03 (8)	-0.14 (9)
N_2_P_2_K_1_	1.06 (6)	1.82 (2)	-1.27 (11)	-0.20 (8)	1.29 (2)	0.62 (4)
N_2_P_2_K_3_	1.35 (4)	0.06 (7)	1.03 (3)	2.34 (1)	-0.62 (10)	0.90 (2)
N_3_P_2_K_2_	1.76 (2)	-4.03 (14)	-1.28 (12)	0.04 (6)	0.69 (5)	-0.42 (10)
N_1_P_1_K_2_	1.62 (3)	2.45 (1)	-0.71 (10)	-1.33 (13)	0.06 (7)	0.71 (3)
N_1_P_2_K_1_	0.13 (8)	1.47 (3)	-1.30 (13)	1.32 (3)	0.93 (3)	0.42 (5)
N_2_P_1_K_1_	-0.63 (11)	-0.12 (9)	1.23 (2)	1.72 (2)	0.80 (4)	0.36 (6)

**Figure 2:**
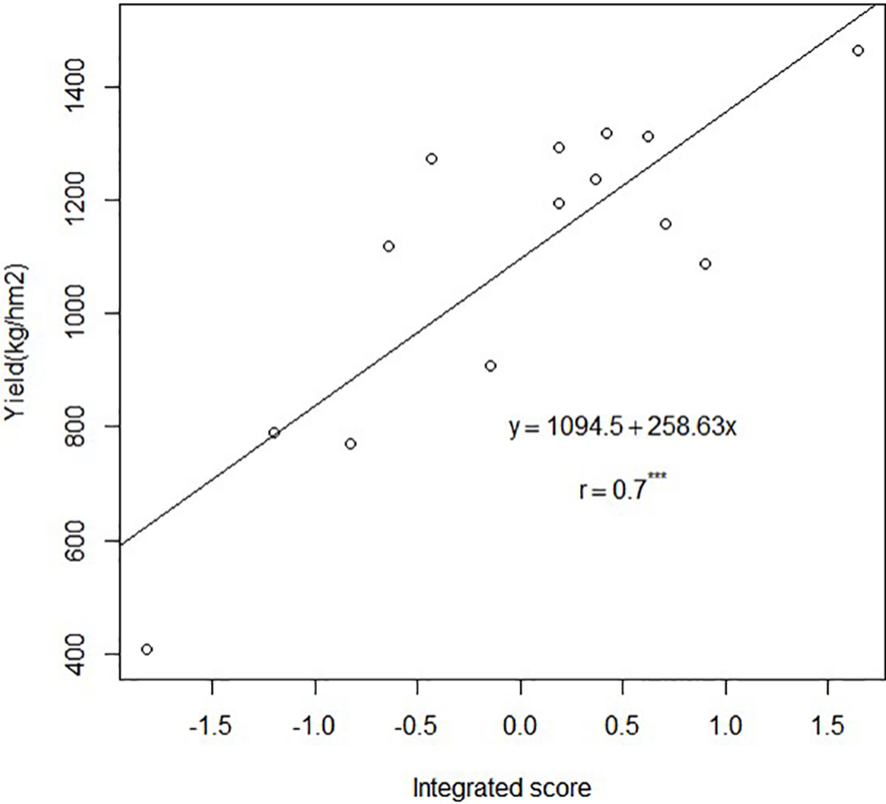
Fit of linear regression between integrated scores and yield of *Sapindus mukorossi* under different fertilization treatments.

The N_2_P_2_K_2_ treatment had the highest integrated scores among all treatments. The integrated scores of the top five treatments were as follows: N_2_P_2_K_2_> N_2_P_2_K_3_> N_1_P_1_K_2_> N_2_P_2_K_1_> N_1_P_2_K_1_. Further correlation analysis showed highly significant positive correlation between integrated scores and *Sapindus mukorossi* yield under different fertilization treatments (**Figure 2**).

### 3.5 Influence of soil and leaf physiological factors on the yield of *Sapindus mukorossi*

The direct and indirect effects of soil and leaf factors on *Sapindus mukorossi* yield were assessed through path coefficient analysis. As shown in **Table 5**, among the soil indicators, soil available nitrogen had the highest direct positive effect on sapodilla yield (0.62), followed by SOC (0.23), and MBC (0.15). These results indicated that soil available nitrogen and SOC were the major factors in increasing crop yield. As for leaf physiological indexes, the direct positive effects on the yield of *Sapindus mukorossi* were in the following order: LAI (0.79) > K (0.37) > ST (0.21) > chlorophyll content (-0.008) > P (-0.03) > SS (-0.13) > N (-0.14). The indirect path coefficients revealed that soil and leaf factors contribute to some extent to the yield of *Sapindus mukorossi* by influencing other factors.

**Table 5:** Direct and indirect path coefficient values of soil and leaf factors on the yield of *Sapindus mukorossi*.

	pH	SOC	AN	AP	AK	MBC	MBN
pH	**-0.11**	0.04	0.06	0.07	0.02	0.05	0.04
SOC	-0.09	**0.23**	0.09	0.14	0.07	0.16	0.18
AN	-0.39	0.25	**0.62**	0.34	0.28	0.12	0.22
AP	0.03	-0.04	-0.03	**-0.06**	-0.01	-0.04	-0.04
AK	0.01	-0.01	-0.02	-0.005	**-0.044**	-0.01	-0.01
MBC	-0.07	0.11	0.03	0.10	0.02	**0.15**	0.07
MBN	-0.04	0.08	0.04	0.07	0.02	0.05	**0.11**
	LAI	SPAD	SS	ST	Nitrogen content	Phosphorus content	Potassium content
LAI	**0.79**	0.25	-0.13	0.35	0.18	0.17	0.06
SPAD	-0.002	**-0.008**	0.001	-0.003	-0.001	-0.005	-0.002
SS	0.02	0.02	**-0.13**	-0.02	-0.05	0.02	-0.05
ST	0.09	0.08	0.03	**0.21**	0.06	0.06	-0.009
Nitrogen content	-0.03	-0.02	-0.05	-0.04	**-0.14**	-0.04	-0.07
Phosphorus content	-0.006	-0.01	0.004	-0.007	-0.008	**-0.03**	-0.01
Potassium content	0.03	0.08	0.14	-0.02	0.21	0.14	**0.37**

Bold numerals are the direct effect values.

### 3.6 Factors influencing soil properties on leaf traits

**Figure 3:**
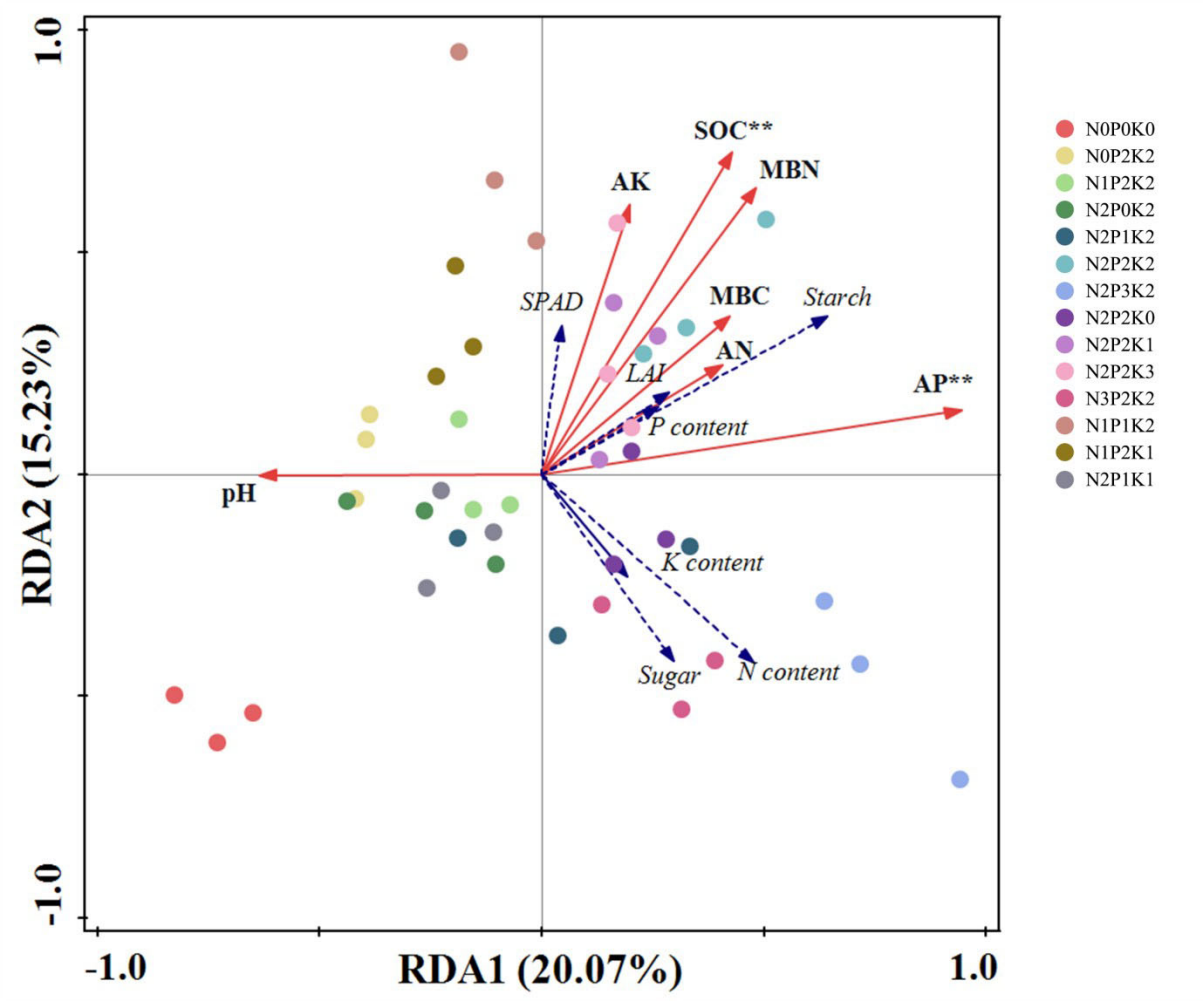
Redundancy analysis (RDA) between soil properties and leaf physiological traits. Soil factors are indicated by solid arrows. Leaf properties are indicated by dashed lines. The first (horizontal) and second (vertical) axes explain 20.07% and 15.23% of the variation. ** means that correlation is significant at the 0.01 level.

To fully assess the relationship between soil properties and leaf traits, we evaluated the effect of soil properties on leaf attributes through RDA analysis (**Figure 3**). The first and second sorting axes explained 35.3% of the total variation. Organic carbon and available phosphorus were closely related to leaf trait attributes. available phosphorus, pH and available nitrogen were more correlated with the first axis, while the second axis was mainly closely related to SPAD soil available potassium. The results showed that organic carbon and effective phosphorus were the two most important soil factors affecting changes in leaf physiological attributes (**Table 6**).

**Table 6:** Revealing the relationship between leaf physiological indicators and soil properties.

indicators	pseudo-F	P
AP	9	0.004
SOC	5.7	0.006
AK	2	0.106
AN	1.8	0.146
MBN	1.8	0.194
pH	0.5	0.61
MBC	0.2	0.85

## 4 Discussion

### 4.1 Effect of fertilization on yield

N, P, and K fertilizers are key elements for high crop yields, and proper application can effectively improve crop yield and quality (Nemadodzi et al., 2017; Xing et al., 2023). It has been shown that there is a close relationship between fertilizers, soil, and plants, and the availability of fertilizers can directly affect soil fertility, which in turn affects plant yield (Chen et al., 2019; Zhang et al., 2023). In this study, it was found that N_2_P_2_K_2_ treatment was the best fertilizer application rate for the combined indexes, and over-fertilization reduced the yield (**Figure 1**, **Table 4**). The possible reason for the increase in yield with the application of N, P and K when the fertilization rate did not reach the threshold was the increase in soil nutrients with the increase in fertilization rate. This was shown by the increase in both soil effective phosphorus and organic carbon content. The path coefficient analysis showed that soil effective phosphorus and organic carbon content played the most important role in increasing the yield of *Sapindus mukorossi*(**Table 5**). This was verified in previous studies (Li et al., 2019; Bindraban et al., 2020). In addition, another reason contributing to the increase in yield may be the increased resistance of the tree itself caused by fertilizer application, the reduction of diseases in the plant and the reduction of insect pests. However, when the rate of fertilizer application exceeds the threshold, fertilizer application can have a negative effect on the plant. Studies in citrus (*Citrus reticulata*) (Li et al., 2019), blueberry (Zhang et al., 2023) and heather (*Phoebe zhennan*) (Yang et al., 2020) also support our finding. The reason for this negative effect may be (1) when the fertilizer application exceeds the threshold, the soil nutrient supply capacity is reduced instead. So, there will be a lower yield because of the weak ability of soil nutrient supply. Another reason is that the higher content of leaves when they are high in nitrogen may imply leaf senescence and delayed growth of other nutrient organs, which leads to consumption of more carbon skeleton and energy by the fruit or storage organs and low carbohydrate accumulation (Li et al., 2019). (2) Excessive fertilizers can be harmful to the environment (Xue et al., 2020). Relevant studies have shown that fertilizer is one of the main sources of soil, water and air pollution. Excessive fertilizers can lead to serious soil sloughing, resulting in the imbalance of soil nutrient structure, which in turn restricts the growth and development of the plant root system, reduces the ability to absorb nutrients and water, and ultimately leads to lower yields. (3) In addition, over-fertilization will also increase plant diseases. For example, studies on diseases of *Panax notoginseng* (Xia et al., 2016), *Zea mays* (Vilela et al., 2022) and *citrus unshiu* (Li et al., 2019) showed that over-fertilization increased the incidence of root rot in *Panax notoginseng* and brown spot in *Coffea species* as well as induced severe defoliation, which resulted in adverse short- and long-term consequences. The present study also found that many new branches sprouted on the whole tree of *Sapindus mukorossi* in high fertilization and the incidence of leaf sooty blotch increased. Therefore, rational application of N, P and K fertilizers will be effective to improve soil and leaf physiological attributes in *Sapindus mukorossi* stands, and play an important role in increasing *Sapindus mukorossi* yield.

### 4.2 Effect of fertilizer application on soil properties

This study showed that the application of nitrogen, phosphorus and potassium had a positive effect on the increase of soil available nutrients. The application of nitrogen, phosphorus and potassium fertilizers increased soil organic carbon and available nitrogen content, but with the increase of nitrogen fertilizers soil organic carbon and available nitrogen content increased firstly and then decreased (**Table 2**). According to the theory of “stoichiometric decomposition” proposed by Hessen et al. (2004), the stoichiometric imbalance of N addition has been shown to accelerate soil organic carbon decomposition in order to maintain the soil C/N, and the decomposition rate and microbial activity are highest when the C and N inputs correspond to the microbial stoichiometric carbon to nitrogen ratio (Hessen et al., 2004). Simultaneous increases in N fertilization accelerated soil organic matter decomposition by increasing the rate of organic carbon decomposition and microbial activity. In energy forests, N additions accompanied by pruning residues (carbon sources) met microbial growth requirements and stimulated mineralization of native soil organic matter and release of reactive N during nitrification, denitrification, and leaching (Chen et al., 2020; Zhao et al., 2020). When excess N fertilizer affects the number and activity of soil microorganisms and the biodegradation of organic carbon sources, it may lead to a reduction in organic carbon content (Ma et al., 2021). Application of excess N fertilizer can lead to soil acidification and reduce soil organic carbon content, with N fertilization (urea application) contributing more to soil acidification than other fertilizers. Urea can be rapidly converted to NH_4_^+^ by soil microorganisms, and during the oxidation of NH_4_^+^, H^+^ is released into the soil. N application leads to the loss of cations (Ca^2+^, K^+^, Mg^2+^) in the soil, which are taken up by plants thereby accelerating soil acidification (Tang et al., 2022, 2023; Wang et al., 2023). Thus, the results illustrate the importance of rational application of N fertilizer in *Sapindus mukorossi* energy woodlands.

The present study also found that the soil available phosphorus content increased gradually with the addition of phosphorus application, indicating that fertilizer application was able to increase the effective phosphorus content in the soil (**Table 2**). This may be due to the fact that fertilizers promoted P-rich microbial activity in the soil, and the addition of phosphorus fertilizers may have resulted in higher levels of residual fast-acting phosphorus nutrients in the soil (Bindraban et al., 2020). The overall trend of increase in total phosphorus, available phosphorus content and total potassium, available potassium content with increase in P and K addition was attributed to the fact that P and K fertilizers themselves contain a large amount of P and K, and the increase in fertilizer application led to more P and K in the soil, which reduces the ability of soil to sequester more P and K. In this study, we found that P addition could increase the available nitrogen content within a certain range, indicating that P addition promoted the mineralization of organic nitrogen and improved the nitrogen uptake and utilization capacity of *Sapindus mukorossi*. Excessive application of potash fertilizer in this study led to a decrease in effective nitrogen and effective phosphorus content, which may be due to the antagonistic effect of phosphate and sulfate ions, related to the high application rate of potassium element (Li et al., 2019). In summary, the appropriate ratio of N, P, and K will increase soil organic carbon, alkaline dissolved nitrogen, and effective phosphorus content, and have obvious effects on soil fast-acting nutrient content, and fertilizer application is an effective way to improve soil nutrients.

### 4.3 Effect of fertilizer application on leaf trait characteristics

Dry matter partitioning is a major determinant of yield formation and involves the yield, total productivity of the plant (Kulmann et al., 2023). The source-sink relationship hypothesis is widely accepted in explaining dry matter production and allocation. The hypothesis proposes three main influencing factors: the competitiveness of sinks, the transport capacity of the phloem, and the supply capacity of the source. Competition between sinks implies that more photo assimilates are preferentially translocated to the fruit during fruit growth and development; the transport capacity of the phloem is closely related to the transport distance; and the supply capacity of the source is closely related to the external environment (fertilization, pruning, canopy). The path coefficient analysis showed that leaf area index, leaf potassium content, and starch content played the most important roles in increasing the yield of *Sapindus mukorossi* (**Table 5**). *Sapindus mukorossi*, as a sun-loving tree species, is significantly affected by canopy microclimate (Zhang et al., 2019). The canopy plays a role in plant growth and development, including respiration and photosynthesis (de Mattos et al., 2023). The distribution of light intensity within the canopy plays a key role in determining photosynthesis throughout its plant canopy, and an overcrowded canopy with poor light intensity has been identified as a major factor in yield decline (Burroughs et al., 2023).

Different fertilization treatments had significant effects on leaf trait characteristics of *Sapindus mukorossi* (**Table 3**). Reasonable application of N, P and K fertilizers can effectively improve the dynamics of leaf nutrient content of plants and significantly increase fruit yield. We found that the addition of N fertilizer significantly improved the leaf physiological traits of *Sapindus mukorossi*, which may be due to the involvement of fertilizer in more physiological and metabolic processes of plant growth (Deng et al., 2019; Lasheen et al., 2021). The results of the study showed that different NPK fertilizer application rates had significant effects on leaf starch and soluble sugars, the total amount of NSCs in *Sapindus mukorossi* leaves increased firstly and then decreased with the application of NPK, the content of soluble sugars increased gradually with the increase of N and P fertilizers, and the content of starch showed an increase firstly and then decreased. Firstly, this may be that more starch was converted to soluble sugar after over-fertilization, which in turn supplemented the carbon demand for plant growth, and physiological processes (Sun et al., 2020). Secondly, at high nitrogen and phosphorus levels, the soluble sugar of the leaves increased significantly, while the starch content decreased significantly, and it is also possible that the soluble sugar of the leaves was more sensitive to high nitrogen and phosphorus treatments, while inhibiting the starch content (Peng et al., 2021). In this study, we also found that K fertilization can promote the absorption of nitrogen in the leaves of *Sapindus mukorossi* N fertilization can increase the potassium content of the leaves. It indicates that leaf physiological processes have to maintain the stoichiometry of nitrogen, phosphorus and potassium, which in turn suggests that the combined application of multiple fertilizers has a more pronounced effect on the physiological metabolism of the leaves than the application of a single type of fertilizer (Guo et al., 2016). The mixed application of nitrogen and potassium fertilizers can improve plant absorption of nitrogen and potassium, nitrogen as an external signal transmitted to the plant root system, the root system to respond to the response, adjusting its own morphological structure to improve the absorption of nitrogen and potassium, and then increase the nitrogen and potassium content in the leaves. At the same time, fertilization can affect leaf chlorophyll synthesis (Xiong et al., 2015). There was no significant difference in chlorophyll content and leaf area index among different fertilization treatments, but the highest chlorophyll content was N_2_P_2_K_2_ treatment. RDA analysis, organic carbon, and effective phosphorus were the two most important soil factors influencing changes in leaf traits (**Figure 3**).

## 5 Conclusions

We demonstrated that fertilization ratio of N, P and K have a great influence on soil properties, leaf physiological traits as well as yield of *Sapindus mukorossi*. Excessive fertilization can negatively affect soil properties and leaf physiological traits. When simultaneously considering the yield, leaf physiological traits and soil fertility, the recommended optimum fertilization ratio for this region are 0.96 kg of N, 0.8 kg of P, and 0.64 kg of K per plant. Soil available nitrogen, soil organic carbon, and leaf area index, were the main factors to improve the crop yield. Soil organic carbon and soil available phosphorus were the most important factors affecting leaf physiological traits. Our results showed that the application of appropriate amounts of N, P, and K fertilizers is an effective way to improve soil fertility and crop yield, and fertilizer application strategies should be developed to meet soil productivity and reduce environmental hazards.

## Data availability statement

The original contributions presented in the study are included in the article/supplementary material. Further inquiries can be directed to the corresponding author.

## Author contributions

JTL: Conceptualization, Data curation, Formal analysis, Funding acquisition, Investigation, Methodology, Project administration, Resources, Software, Supervision, Validation, Visualization, Writing – original draft, Writing – review & editing. DW: Conceptualization, Data curation, Formal analysis, Investigation, Methodology, Software, Validation, Writing – review & editing. XY: Formal analysis, Methodology, Supervision, Writing – review & editing. LJ: Conceptualization, Data curation, Formal analysis, Funding acquisition, Investigation, Methodology, Project administration, Resources, Software, Supervision, Validation, Visualization, Writing – original draft, Writing – review & editing. NC: Data curation, Formal analysis, Supervision, Writing – review & editing. JJL: Data curation, Funding acquisition, Methodology, Resources, Supervision, Writing – review & editing. PZ: Conceptualization, Formal analysis, Funding acquisition, Methodology, Resources, Supervision, Software, Validation, Writing – review & editing. LZ: Conceptualization, Funding acquisition, Methodology, Resources, Project administration, Supervision, Writing – review & editing. QC: Funding acquisition, Methodology, Resources, Supervision, Formal analysis, Investigation, Validation, Writing – review & editing.
